# Remarkable Active Site Utilization in Edge‐Hosted‐N Doped Carbocatalysts for Fenton‐Like Reaction

**DOI:** 10.1002/advs.202404958

**Published:** 2024-09-11

**Authors:** Huajie Zhong, Zeyu Gong, Jiaxing Yu, Yu Hou, Yuan Tao, Qi Fu, Huangsheng Yang, Xinzhe Xiao, Xingzhong Cao, Junhui Wang, Gangfeng Ouyang

**Affiliations:** ^1^ School of Chemical Engineering and Technology Sun Yat‐Sen University Zhuhai Guangdong 519082 P. R. China; ^2^ MOE Key Laboratory of Bioinorganic and Synthetic Chemistry/KLGHEI of Environment and Energy Chemistry School of Chemistry Sun Yat‐Sen University Guangzhou Guangdong 510275 P. R. China; ^3^ Institute of High Energy Physics Chinese Academy of Sciences Beijing 100049 P. R. China; ^4^ College of Chemistry & Molecular Engineering Center of Advanced Analysis and Computational Science Zhengzhou University Zhengzhou 450001 P. R. China; ^5^ Guangdong Provincial Key Laboratory of Emergency Test for Dangerous Chemicals Guangdong Institute of Analysis (China National Analytical Center Guangzhou) Guangdong Academy of Science 100 Xianlie Middle Road Guangzhou 510070 P. R. China

**Keywords:** atom utilization, carbon defect, carbon nanomaterials, Fenton‐like reaction

## Abstract

Improving the utilization of active sites in carbon catalysts is significant for various catalytic reactions, but still challenging, mainly due to the lack of strategies for controllable introduction of active dopants. Herein, a novel “Ar plasma etching‐NH_3_ annealing” strategy is developed to regulate the position of active N sites, while maintaining the same nitrogen species and contents. Theoretical and experimental results reveal that the edge‐hosted‐N doped carbon nanotubes (E‐N‐CNT), with only 0.29 at.% N content, show great affinity to peroxymonosulfate (PMS), and exhibit excellent Fenton‐like activity by generating singlet oxygen (^1^O_2_), which can reach as high as 410 times higher than the pristine CNT. The remarkable utilization of edge‐hosted nitrogen atom is further verified by the edge‐hosted‐N enriched carbocatalyst, which shows superior capability for 4‐chlorophenol degradation with a turnover frequency (TOF) value as high as 3.82 min^−1^, and the impressive TOF value can even surpass those of single‐atom catalysts. This work proposes a controllable position regulation of active sites to improve atom utilization, which provides a new insight into the design of excellent Fenton‐like catalysts with remarkable atom utilization efficiency.

## Graphical abstract

To identify the effective defect for improving the utilization of nitrogen atom, the controllable regulation strategy in N‐doped carbon is desirable. Herein, an “Ar plasma etching‐NH_3_ annealing” strategy is presented to deliberately regulate the nitrogen sites, while maintaining the same nitrogen species and contents. Various results indicate edge‐hosted N sites present higher performance to activate PMS for generating singlet oxygen.

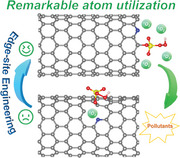

## 1 Introduction

Carbon‐based metal‐free catalysts (CMFCs) are generally regarded as one kind of the most attractive catalysts applied in various fields, including energy conversion and storage,^[^ ^1^ ^]^ fluorescent sensing.^[^ ^2^ ^]^ and environmental remediation,^[^ ^3^ ^]^ mainly due to their unique advantages, including low cost, tolerance to both alkaline and acid conditions, high electronic conductivity, and controllable atomic/molecular structures.^[^ ^4^ ^]^ In order to develop cost‐effective and high‐performance CMFCs, various strategies have been proposed, such as heteroatom doping.^[^ ^5^ ^]^ and intrinsic defect constructing.^[^ ^6^ ^]^ Among them, nitrogen doping is one of the most efficient and economic strategies,^[^ ^7^ ^]^ which could tailor the electron structure and/or introduce substitutional defects to the carbon matrix, thus improving the chemical activities and/or increasing the number of active sites.^[^ ^8^ ^]^ Therefore, tremendous efforts have been made to develop carbocatalysts with high nitrogen content via tedious synthetic procedures.^[^ ^9^ ^]^ However, due to the burial of a certain amount of N dopants within the dense carbon substrate, the active sites cannot be fully utilized. Moreover, because of the tight distribution of nitrogen atoms, different types of N sites would interfere with each other, leading to significantly diminished catalytic activities.^[^ ^10^ ^]^ Thus, the introduction of excessive nitrogen species can result in low nitrogen atom utilization efficiency and turnover frequency (TOF).

Recently, it has been realized that reasonable regulation of the intrinsic defects (e.g., edge, vacancy) in carbon matrix could greatly enhance the catalytic activity of the dopant‐free carbocatalysts, since they can induce the asymmetric local redistribution of electron density.^[^ ^11^ ^]^ Moreover, theoretical and experimental investigations indicated that N dopants and intrinsic defects could induce a synergistic effect on promoting the catalytic activity of carbocatalysts.^[^ ^12^ ^]^ Thus, it can be inferred that the nitrogen atom utilization efficiency could be enhanced by tailoring the intrinsic defects adjacent to it. Although nitrogen species can be doped in the defective carbon via different strategies,^[^ ^13^ ^]^ the positions of doped nitrogen atoms are random and the nitrogen species are uncontrollable.^[^ ^14^ ^]^ Therefore, to identify the effective defect for improving the utilization of nitrogen atom, the controllable regulation strategy of the position of nitrogen species (N dopants adjacent to different intrinsic carbon defects) in metal‐free N‐doped carbon is highly desirable, yet still challenging.

Herein, we present an “Ar plasma etching‐NH_3_ annealing” strategy to introduce nitrogen atoms at different defective sites (edge and vacancy) in graphitized multi‐walled carbon nanotubes, while maintaining the same nitrogen species and contents (namely E‐N‐CNT and V‐N‐CNT). The materials were applied as Fenton‐like catalysts to activate peroxymonosulfate (PMS) for organic pollutants degradation. Theoretical and experimental results indicate that edge‐hosted N sites present much higher catalytic activity to activate PMS for the generation of reactive species (singlet oxygen, ^1^O_2_). As a result, the optimized edge‐hosted N‐enriched carbocatalyst exhibits impressive catalytic performance with a TOF value as high as 3.82 min^−1^ for 4‐chlorophenol (4‐CP) degradation, and the remarkable TOF value could even match up with those of single‐atom catalysts. Our work not only provides a promising strategy for the controllable fabrication of N dopants on different intrinsic carbon defects, but also illustrates that edge‐site engineering could greatly enhance the nitrogen atom utilization efficiency for Fenton‐like reactions.

## 2 Results and Discussion

### 2.1 Synthesis and Characterization of Controllable N‐Doped Carbon Nanotubes

To investigate the probability of controllable nitrogen doping on two major intrinsic defects in carbon materials (edge and vacancy defects), density function theory (DFT) calculations were first used to explore the N doping mechanism. As shown in Figure S1, Supporting Information; N dopants (pyridinic N) are energetically favorable on the edge and vacancy defects (the formation energies are −2.16 and −1.96 eV, respectively), which suggests that pyridinic N doping could be realized on the edge and vacancy defects. Moreover, the formation energy of pyridinic N at the edge defects is obviously more negative than that at the vacancy defects, indicating that the N atoms have a preference to situate at the edge defects of the carbon matrix. Thus, it can be inferred that, through the intentional design of vacancy‐rich and edge‐rich carbon structures, controllable N doping could be achieved.

**Figure 1:**
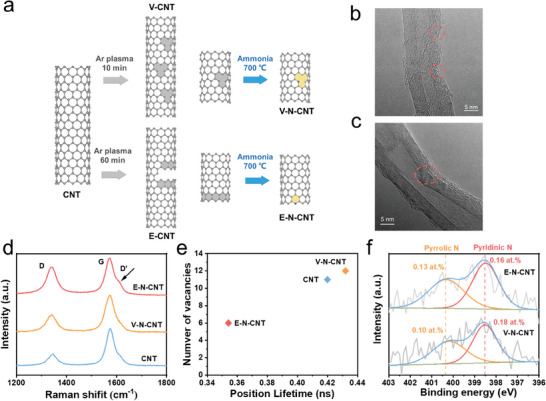
Synthetic scheme and characterization of E‐N‐CNT and V‐N‐CNT. a) Illustration of the preparation of E‐N‐CNT and V‐N‐CNT. b) HRTEM image of V‐N‐CNT. c) HRTEM image of E‐N‐CNT. d) Raman spectra of E‐N‐CNT, V‐N‐CNT and CNT. e) Calculated positron lifetimes (*τ_2_*) of different vacancy‐type defects with plane symmetries of vacant sites. f) The N 1s high‐resolution XPS spectra of E‐N‐CNT and V‐N‐CNT.

Plasma etching has been reported as a controllable method to induce different types of defects in carbon materials by regulating treatment time, input power, and frequency while keeping the integrity of the bulk structure.^[^ ^15^ ^]^ In particular, argon (Ar) plasma etching is more feasible for producing exposed sites with hexagon carbons,^[^ ^15a^ ^]^ thus vacancy defects and edge defects could be preferentially created. Moreover, compared with other plasma sources (like N_2_ and air), the inert Ar^+^ does not bond to the carbon matrix.^[^ ^16^ ^]^ and introduce extra heteroatoms (e.g., oxygen group or nitrogen dopant), which is favorable to the following N doping process to achieve controllable N species and contents. Therefore, the Ar plasma was selected to create vacancy defects and edge defects in the graphitized multi‐walled carbon nanotubes (with much lower initial defects). The vacancy‐rich carbon nanotubes (V‐CNT) could be realized with a short plasma exposure time. To obtain edge‐rich carbon nanotubes (E‐CNT), a longer etching time is needed.^[^ ^17^ ^]^ After the Ar plasma etching, thousands of docking sites at the defective region were created, which were more easily replaced by nitrogen atoms.^[^ ^18^ ^]^ Subsequently, the V‐CNT and E‐CNT were further annealed at 700 °C in the ammonia (NH_3_) atmosphere to dope with nitrogen (V‐N‐CNT and E‐N‐CNT) (**Figure** **1**a).^[^ ^7b^ ^]^

The XRD patterns of the materials are shown in Figure S2 (Supporting Information), and all the patterns displayed two broad peaks ≈25.5° and 42.6°, corresponding to the (002) and (101) planes of graphite.^[^ ^9c^ ^]^ And a notable reduction in the intensity could be detected for E‐N‐CNT and V‐N‐CNT compared to CNT, suggesting that more defects were produced by plasma etching.^[^ ^13^, ^19^ ^]^ Figure S3 (Supporting Information) shows the near‐edge X‐ray absorption fine structure of the catalysts. The peak located at ≈285.4 was ascribed to the C 1s → π* and σ* transition,^[^ ^20^ ^]^ and the lower intensity of π* of E‐N‐CNT was observed, suggesting the higher carbon defects in E‐N‐CNT. The high‐resolution transmission electron microscope (HRTEM) was applied to straightforwardly and visually demonstrate the formation of specific defects. After the plasma treatment in a short time, the tubes of CNT were damaged and vacancy defects could be clearly found in V‐N‐CNT (Figure 1b). As a result of prolonged exposure to argon irradiation, the carbon nanotubes were cracked, and more edge defects were discovered in E‐N‐CNT (Figure 1c). The process of plasma etching can be well illustrated by the observations of HRTEM images (Figure S4, Supporting Information). It can be observed that there were still some vacancy defects on the E‐N‐CNT, indicating that edge defects were derived from vacancies, which were deeply fractured during the prolonged exposure time. To further verify the type of defects, Raman spectra of different carbon nanotubes were measured. Especially, the signal of D’ peak in Raman spectra is sensitive to the boundaries (edges) in graphene.^[^ ^21^ ^]^ It could be clearly observed that, compared with the negligible detected D’ peak at 1610 cm^−1^ in CNT and V‐N‐CNT, the D’ signal became obvious in E‐N‐CNT (Figure 1d), indicating the introduction of edge defects after the argon plasma treatment time reached 60 min. Furthermore, positron annihilation lifetime spectroscopy (PALS) has also been applied to confirm the types of defects in the CNT samples (Figure S5, Supporting Information). The PALS spectra were divided into three‐lifetime ingredients (*τ_1_*, *τ_2_*, and *τ_3_*) (Table S1, Supporting Information). The medium lifetime component (*τ_2_*) is attributed to the type of various V_x_ defects (x‐vacancy, represents the absence of x neighboring carbon in the vacancy) (Figure 1e).^[^ ^22^ ^]^ By analyzing the PALS lifetimes, it could be found that after plasma etching of 10 min, one carbon atom was removed from the carbon matrix and vacancy defects were created in V‐N‐CNT. In contrast, *τ_2_* was greatly decreased in the E‐N‐CNT, since the vacancies were destroyed and converted into edges under the strong and persistent attack of plasma, resulting in fewer vacancies being available to capture the positron. These results suggested that when the treatment time was 10 min, more vacancy defects were produced in V‐N‐CNT. However, when the treatment time reached 60 min, the vacancies became larger and the etching sites became deeper, finally causing the fracture of CNT and more edge defects to appear in E‐N‐CNT. Thus, vacancy‐rich and edge‐rich carbon nanotubes were obtained by controllable Ar plasma etching.

Furthermore, the N contents and configurations were identified by the X‐ray photoelectron spectroscopy (XPS) (Figure 1f). The V‐N‐CNT and E‐N‐CNT were dominated by pyridinic nitrogen (398.5 eV) and pyrrolic nitrogen (400.5 eV), without graphitic N (401 eV), implying N atoms were successfully doped at the defects instead of the inner plane of CNT after Ar plasma etching.^[^ ^23^ ^]^ Notably, the N configurations and their corresponding contents were nearly the same for E‐N‐CNT (including 0.16 at. % of pyridinic N and 0.13 at.% of pyrrolic N) and V‐N‐CNT (including 0.18 at.% of pyridinic N and 0.10 at.% of pyrrolic N), indicating the controllable synthesis of N‐doped carbon nanotubes were achieved by the “Ar plasma etching‐NH_3_ annealing” strategy.

### 2.2 Fenton‐like Activities of N‐Doped Carbon Nanotubes

**Figure 2:**
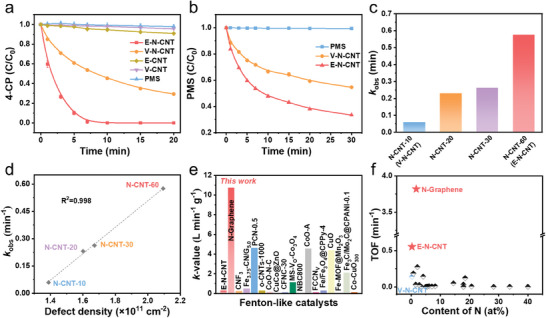
Catalytic performance for pollutant degradation. a) 4‐CP degradation efficiency. b) PMS decomposition efficiency. c) The rate constants (*k*_obs_) of N‐CNTs. d) Correlation between *k*_obs_ and defect. e) Comparison of the catalytic efficiencies of 4‐CP removal by PMS activation with different catalysts. f) Comparison of the nitrogen atom utilization efficiency of pollutant removal by PMS activation with different N‐hosted metal‐free carbon catalysts. Reaction conditions: catalyst dosage = 0.1 g L^−1^, [PMS] = 1.5 mm, [4‐CP] = 10 ppm.

The catalytic performances of the CNT samples for Fenton‐like reactions were evaluated by activation of PMS for 4‐CP degradation. Although E‐CNT showed a high ratio of *I_D_/I_G_* (Figure S6, Supporting Information), the 4‐CP removal efficiency was less than 10% in E‐CNT/PMS systems, reflecting that the intrinsic defects were nearly unable to activate PMS (**Figure** **2**a). The degradation ratio of 4‐CP was ≈70% in 20 min in V‐N‐CNT/PMS system, with a reaction rate constant (*k_obs_*) of 0.0595 min^−1^ (Figure S8, Supporting Information). Surprisingly, almost 100% removal of 4‐CP was achieved by E‐N‐CNT in 7 min, with greatly higher *k_obs_* (0.5755 min^−1^), which is as high as 10 times higher than that of V‐N‐CNT, and 410‐fold of pristine CNT. Additionally, the specific surface area of E‐N‐CNT was similar to that of V‐N‐CNT (Figure S9, Supporting Information), excluding the contribution of adsorption process to 4‐CP degradation. And only 15% 4‐CP was adsorbed by E‐N‐CNT, implying that the removal of 4‐CP was mainly due to the oxidation reaction (Figure S10, Supporting Information). To exclude the impact of the annealing process on the catalyst, E‐CNT was annealed at the same temperature in Ar atmosphere for 3 h to produce E‐CNT‐700. It could be observed that E‐CNT‐700 exhibited poor catalytic activity (20% 4‐CP removal in 20 min) (Figure S7, Supporting Information), indicating the important role of edge‐hosted nitrogen sites. Furthermore, PMS consumption experiments were conducted to compare the efficiencies of PMS activation between E‐N‐CNT and V‐N‐CNT. As shown in Figure 2b, the consumption of PMS is higher in the E‐N‐CNT/PMS system, which is consistent with the increased *k_obs_* for the degradation of 4‐CP. Moreover, as shown in Table S2 (Supporting Information), E‐N‐CNT presents a nearly 4 times higher TOF value (min^−1^) for pollutant degradation than that of V‐N‐CNT, indicating that the position of the active N species is determinative to the catalytic performance.

To better understand the importance of the position of the active N site, a series of N‐doped CNTs (N‐CNT‐x, x represents the etching time) were obtained by the “Ar plasma etching‐NH_3_ annealing” strategy. Raman spectra were first performed to quantify the defect densities of the N‐CNTs (Figure S11, Supporting Information). As expected, the disordered defect was increasing along with prolonged plasma etching, which was consistent with the mechanism of plasma etching. The increasing defect density indicated that the “activated area” (attacked by plasma etching) has gradually coalesced and more edge defects would be dominated in CNTs.^[^ ^24^ ^]^ Moreover, the XPS spectra of N‐CNTs were obtained, and nearly the same nitrogen species and contents were presented in various N‐CNTs (Figure S12, Supporting Information; Figure 1f). The above results indicated that more edge‐hosted nitrogen sites could be produced with the increasing etching time. Furthermore, the Fenton‐like activities of N‐CNTs were evaluated (Figure S13, Supporting Information). The 4‐CP degradation kinetics was further fitted by the pseudo‐first‐order model to get a clear comparison of the activities (Figure 2c). Notably, it could be found that the catalytic activity was significantly improved with increasing edge defect intensity while keeping the same N dopants. As shown in Figure 2d, the *k*_obs_ is positively correlated with defect density with a linear correlation (R^2^ = 0.998), revealing that edge‐hosted nitrogen sites are more capable of stimulating PMS activation than vacancy‐hosted nitrogen sites.

Furthermore, as a model carbon material with rich edge defects, graphene was selected to verify the excellent performance of PMS activation by edge‐hosted nitrogen atoms.^[^ ^25^ ^]^ N doping graphene (N‐Graphene) was prepared by NH_3_ annealing to import edge‐hosted nitrogen. As shown in Table S4 (Supporting Information), the N 1s XPS spectra of N‐Graphene displayed significantly higher pyridinic N (0.79 at%) and pyrrolic N (0.47 at%), which are ascribed as edge‐type N,^[^ ^26^ ^]^ than those of Graphene. The edge‐type N was also identified through the energy‐dispersive X‐ray spectroscopy (EDS) mapping, and the white line representing the distribution of nitrogen atoms along the edge (Figure S16, Supporting Information). Not surprisingly, N‐Graphene displayed remarkable catalytic performance, and the pollutant was rapidly degraded in just 10 s (Figure S17, Supporting Information), exhibiting unprecedented performance with *k*_obs_ even reaching 20.116 min^−1^. Moreover, *k*‐value was calculated to compare the catalytic performance of N‐Graphene and the typical reported Fenton‐like catalysts (Table S3, Supporting Information), and N‐Graphene exhibited the highest *k*‐value (as high as 10.73 L min^−1^ g^−1^) (Figure 2e), further revealing that the edge‐hosted nitrogen atom is the efficient active site. Furthermore, the utilization efficiencies of nitrogen atoms in N‐doped metal‐free carbon catalysts in Fenton‐like reactions were also concluded (Figure 2f). Although the contents of nitrogen atoms in E‐N‐CNT and N‐Graphene are not rich, their TOF values are remarkably greater than other N‐doped metal‐free carbon catalysts, reflecting the remarkable utilization efficiencies of edge‐hosted nitrogen atoms in PMS activation.

### 2.3 Investigation of the Catalytic Mechanism of E‐N‐CNT/PMS Systems

**Figure 3:**
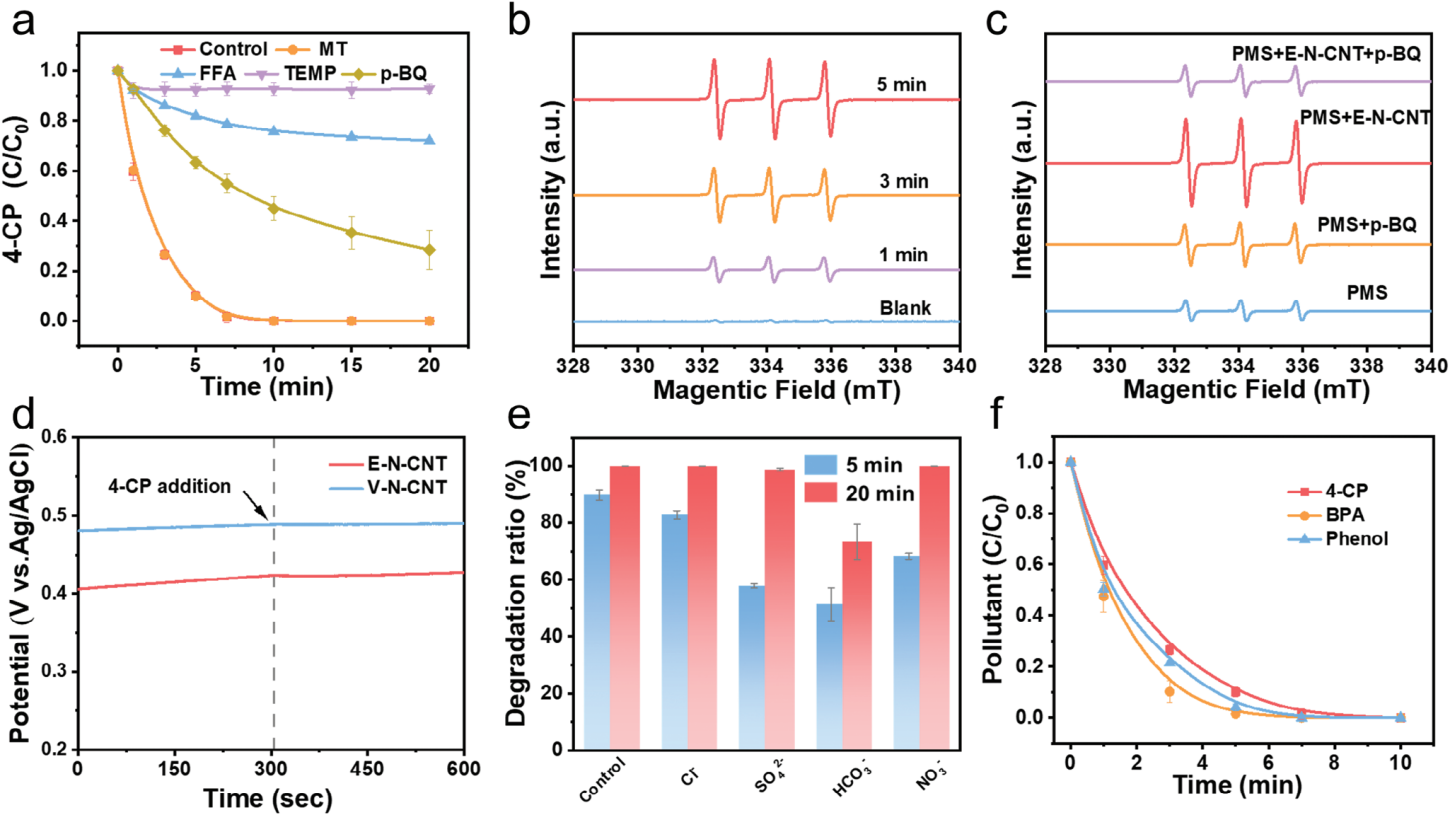
Mechanism investigation of E‐N‐CNT/PMS system. a) 4‐CP degradation efficiency under different quenching conditions. b,c) EPR spectra of TEMP in E‐N‐CNT/PMS system (water as the solvent). d) Changes in open‐circuit potential of the E‐N‐CNT‐PMS complex and V‐N‐CNT‐PMS complex after the addition of 4‐CP. e) The degradation ratio of 4‐CP degradation in the presence of various inorganic anions. f) Degradation curves of different pollutants in the E‐N‐CNT/PMS system. Reaction conditions: catalyst dosage = 0.1 g L^−1^, [PMS] = 1.5 mm, [4‐CP] = 10 ppm, [MT] = 750 mm, [p‐BQ] = [FFA] = [TEMP] = 7.5 mm, [BPA] = 20 ppm, [Phenol] = 10 ppm, [Cl^−^] = [SO_4_^2−^] = [HCO_3_^−^] = [NO_3_^−^] = 10 mm.

To identify the catalytic mechanism of the E‐N‐CNT/PMS system, a series of experiments were performed. First, the quenching tests were conducted to investigate the possible mechanism (**Figure** **3**a). Methanol (MT) is a quenching agent of ·OH and SO_4_·^−^, furfuryl alcohol (FFA) and tetramethylpiperidine (TEMP) were selected as the singlet oxygen (^1^O_2_) quenchers, and p‐benzoquinone (p‐BQ) was utilized to capture O_2_^·−^.^[^ ^26^ ^]^ The E‐N‐CNT/PMS system still achieved the great 4‐CP removal when MT was added, suggesting the absence of ·OH and SO_4_·^−^ during the activation of PMS. However, the addition of p‐BQ inhabited the removal efficiency of 4‐CP in E‐N‐CNT/PMS system, indicating the presence of O_2_^·−^. Additionally, when FFA was applied to quench ^1^O_2_, the degradation rate dropped sharply from 0.5575 to 0.0147 min^−1^ (Figure S19, Supporting Information), and only 7% 4‐CP was degraded in the presence of TEMP, reflecting that ^1^O_2_ made a vital contribution to the high 4‐CP degradation.

The electron paramagnetic resonance (EPR) experiments were further conducted to investigate the possible catalytic mechanism in the E‐N‐CNT/PMS system. 5,5‐dimethyl‐1‐pyrroline (DMPO) and TEMP were individually used as the spin traps of radicals and ^1^O_2_. As shown in Figure S20 (Supporting Information), a typical seven‐line EPR signal of 5,5‐dimethyl‐1‐pyrrolidone‐N‐oxyl (DMPOX) was dominant in the E‐N‐CNT/PMS system. The formation of DMPOX is related to DMPO oxidation by ·OH, high‐valent metals, or ^1^O_2_.^[^ ^27^ ^]^ Combined with the quenching results, the addition of MT exhibited a slight inhibition of 4‐CP degradation, implying the mechanism of E‐N‐CNT/PMS system was absolutely a nonradical pathway. Moreover, there was a strong characteristic signal of TEMP‐^1^O_2_ with triple peaks in the E‐N‐CNT/PMS system (Figure 3b), unveiling the generation of ^1^O_2_. However, the intensity of the TEMP‐^1^O_2_ signals decreased after the addition of 4‐CP, confirming the important role of ^1^O_2_ in the degradation of 4‐CP (Figure S21). Furthermore, DMPO was applied in the methanol solution to detect the production of O_2_^·−^, because the generated O_2_^·−^ would facilitate the generation of ^1^O_2_,^[^ ^28^ ^]^ and p‐BQ could decrease the 4‐CP degradation rate in the E‐N‐CNT/PMS system. The signal of DMPO‐O_2_^·−^ was recorded with multiple peaks (Figure S22, Supporting Information), implying the existence of O_2_^·−^. To clarify the relationship between ^1^O_2_ and O_2_^·−^, p‐BQ was added to the E‐N‐CNT/TEMP system. The intensity of TEMP‐^1^O_2_ signal greatly dropped after the addition of p‐BQ (Figure 3c), revealing that the reactive species ^1^O_2_ was generated from O_2_^·−^. In addition, in situ measurements of open circuit potential were utilized to identify the existence of the electron transfer process (ETP). When 4‐CP was injected into the solution (N‐CNT and PMS), the potentials showed a negligible decline (Figure 3d), excluding the participation of ETP in the E‐N‐CNT/PMS system.^[^ ^29^ ^]^ Therefore, ^1^O_2_ is dominated in the E‐N‐CNT/PMS system, instead of ETP and radicals. Interestingly, the intensity of the TEMP‐^1^O_2_ signal detected in the E‐N‐CNT/PMS system was stronger than that in V‐N‐CNT/PMS system, which corresponds to the superior activities of PMS activation to degrade 4‐CP (Figure S23, Supporting Information).

It is well known that radicals would be inevitably consumed by the coexisting inorganic ions, which greatly weaken their reaction performance with target pollutants.^[^ ^30^ ^]^ However, ^1^O_2_ is less likely to be affected by the inorganic ions.^[^ ^31^ ^]^ As shown in Figure 3e, there was no obvious inhibition on the removal of 4‐CP with the involvement of inorganic anions such as SO_4_^2−^, Cl^−^, NO_3_^−^ and HCO_3_^−^ (Figure S24, Supporting Information), implying the E‐N‐CNT/PMS system with ^1^O_2_ can successfully resist the disturbance of background ions. The excellent performance was further verified by other organic pollutants. All the pollutants could be degraded completely in only 10 min, showing the superior removal efficiencies of the E‐N‐CNT/PMS system (Figure 3f).

The stability of E‐N‐CNT was also investigated, and as shown in Figure S25 (Supporting Information), the used catalyst exhibited reduced activity. XPS, Raman, and TEM analyses were further conducted to investigate the origin of the poor cycle stability. As shown in Table S5 (Supporting Information), the content of O atoms of E‐N‐CNT showed no change after treatment by PMS, which suggested that the self‐oxidation of E‐N‐CNT by PMS was negligible. Moreover, nearly no changes in N species, morphologies, and defect densities of the used E‐N‐CNT were observed (Figures S26 and S27, Table S4, Supporting Information), demonstrating that E‐N‐CNT remained stable during the reaction. However, as shown in Table S5 (Supporting Information), the content of oxygen has been increased and the element of chlorine was detected in the used‐E‐N‐CNT based on the XPS analysis, suggesting the surface accumulation of 4‐CP degradation products, which resulted in the poor cycle stability.^[^ ^32^ ^]^ To remove the adhesion, thermal treatment in Ar atmosphere was conducted, and as expected, the catalyst could be regenerated and achieve superior 4‐CP removal efficiency (Figure S25, Supporting Information).

### 2.4 Structural Origin of N‐CNTs for PMS Activation

**Figure 4:**
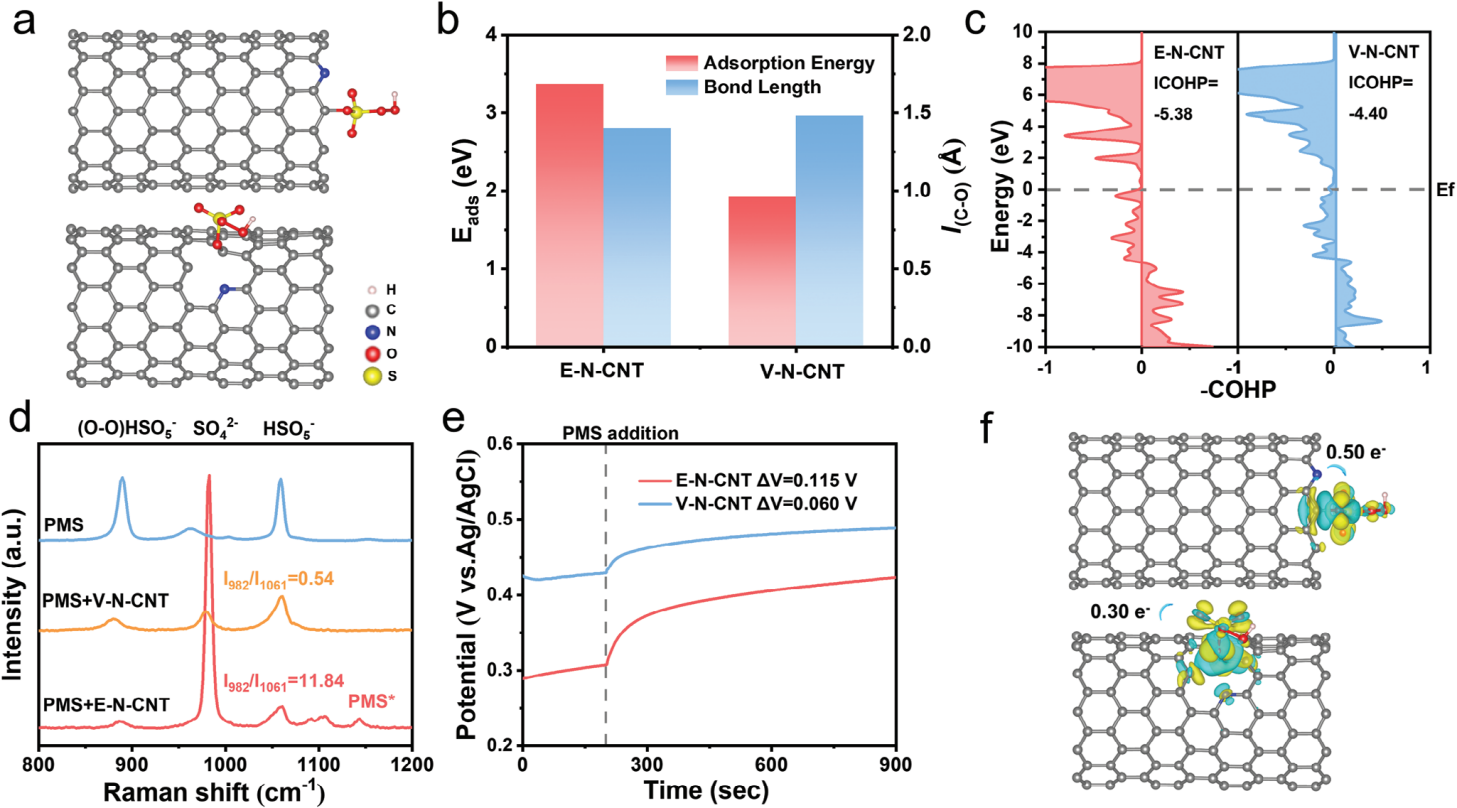
Theoretical and experimental verification of N‐CNTs for PMS activation. a) Adsorption model of PMS on E‐N‐CNT (upside) and V‐N‐CNT (downside). b) Adsorption energies of PMS on the C sites of E‐N‐CNT and V‐N‐CNT and the lengths of C─O bond. c) The COHP between N‐CNTs and PMS. d) In situ Raman spectra of PMS, V‐N‐CNT/PMS and E‐N‐CNT/PMS system. e) Changes in open‐circuit potential of the E‐N‐CNT‐PMS complex and V‐N‐CNT‐PMS complex. f) Differential charge of E‐N‐CNT and V‐N‐CNT after adsorption of PMS molecule, and the corresponding charge transfer. The cyan and yellow regions represent areas of electron deficiency and areas of increased electron density, respectively.

DFT calculations were conducted to obtain theoretical insights into the possible interaction between N‐CNTs and PMS. According to the structural analyses of E‐N‐CNT and V‐N‐CNT, two optimized models were constructed for the calculations (Figures S28 and S29, Supporting Information), and the corresponding PMS adsorption energies (E_ads_) on E‐N‐CNT and V‐N‐CNT were also calculated. Two possible adsorption sites including N site and the neighboring C site were considered (**Figure** **4**a; Figure S30, Supporting Information), since the introduction of N atoms could regulate the charge distribution in the local carbon matrix, resulting in neighboring C becoming the possible active site.^[^ ^9b^ ^]^ The PMS adsorption energies are −3.37 and −1.92 eV on the C site of E‐N‐CNT and V‐N‐CNT, respectively. In contrast, the PMS adsorption energies are decreased to −0.91 eV (E‐N‐CNT) and −0.60 eV (V‐N‐CNT) at the N site (Figure S31, Supporting Information). The higher adsorption energies on the C site confirm that the possible active site is the C atom adjacent to N. Moreover, the adsorption energy to E‐N‐CNT is much higher than that to V‐N‐CNT, revealing that the interaction between PMS molecule and E‐N‐CNT is stronger, which further elucidates the excellent PMS activation performance of E‐N‐CNT.

Furthermore, in order to better understand the interaction between PMS molecules and N‐CNTs, the C─O bond was analyzed (Figure 4b). The length of C─O bond of E‐N‐CNT (1.404 Å) is much shorter than that of V‐N‐CNT (1.480 Å), suggesting the profound interaction between PMS molecules and E‐N‐CNT. Moreover, the crystal orbital Hamilton population (COHP) of E‐N‐CNT and V‐N‐CNT was established to compare the interaction between N‐doped catalysts and PMS (Figure 4c), and the integrated COHP (ICOHP) value representing the bond strength was calculated. The ICOHP values were calculated to be −5.38 and −4.40 for E‐N‐CNT and V‐N‐CNT, respectively, reflecting the edge‐hosted nitrogen atoms greatly improve the interaction between the active site and PMS.^[^ ^33^ ^]^ Furthermore, the stronger interaction between PMS and E‐N‐CNT was verified by experiments. The in situ Raman spectra were recorded to analyze the interaction between N‐CNTs and PMS. As shown in Figure 4d, the pure PMS solution exhibits three main peaks at 876, 982, and 1061 cm^−1^ corresponding to the stretching vibrations of O‐O, SO_4_^2−^ and SO_3_^−^ in free PMS (H‐O‐O‐SO_3_^−^), respectively.^[^ ^34^ ^]^ In the presence of E‐N‐CNT, a new peak of PMS* (1143 cm^−1^) was detected in the spectrum of the E‐N‐CNT /PMS system.^[^ ^35^ ^]^ The value of I_982_/I_1061_ was further measured for the N‐CNTs/PMS system, which could be regarded as an indicator for the consumption of PMS, and a higher value suggested the greater consumption of PMS.^[^ ^32^ ^]^ Notably, the I_982_/I_1061_ ratio increased from 0.54 to 11.84 when comparing V‐N‐CNT with E‐N‐NCT. To better reveal the reaction dynamics, in situ Raman spectra over time were conducted. As shown in Figure S32 (Supporting Information), the ratio of I_982_/I_1061_ continuously rises over time, suggesting the rapid decomposition of HSO_5_^−^ to SO_4_^2−^ by E‐N‐CNT, revealing that the active site at edge promoted the optimal PMS adsorption and decomposition. This result is also consistent with the PMS consumption experiment as shown in Figure 2b. Moreover, the robust interaction between PMS molecular and E‐N‐NCT is corroborated by in situ measurements of open circuit potential of PMS* complex on catalysts (Figure 4e). After the addition of PMS, the open circuit potentials increased, reflecting the formation of PMS* with enhanced oxidation power. The potentials increased higher for E‐N‐CNT‐PMS (0.115 V) than for V‐N‐CNT‐PMS (0.060 V), indicating the stronger PMS adsorption ability of E‐N‐CNT.^[^ ^36^ ^]^

Additionally, to illustrate the electron transfer between PMS molecules and N‐CNTs, the differential charges of the E‐N‐CNT/PMS and V‐N‐CNT/PMS system were calculated. As shown in Figure 4f, the increasing electron densities of PMS suggest that N‐CNTs inject their electrons into PMS. Moreover, Bader charge calculations reveal that edge‐hosted N enhances the charge transfer from the C site to PMS (0.50 e) compared to vacancy‐hosted N (0.30 e). The increasing charge transfer demonstrates the remarkable electronic rearrangement between E‐N‐CNT and PMS, resulting in the generation of PMS* complex,^[^ ^33^ ^]^ and consequently promoting the generation of ^1^O_2_ in the E‐N‐CNT/PMS system. The direction of electron transfer was verified by the in situ Raman spectra (Figure S33, Supporting Information), in which the obvious blueshift of the D peak suggested that the carbon catalyst is electron deficient.^[^^37^^]^

In conclusion, the calculation results indicate that the position of the nitrogen atom is vital for the carbon catalyst to efficiently activate PMS. It could enhance the interaction between active sites (C atoms) and PMS molecules and promote electron transport from E‐N‐CNT to PMS, thus leading to the superior activity of E‐N‐CNT for PMS activation.

## 3 Conclusion

In summary, we proposed a strategy for the precise regulation of nitrogen doping on defective carbons, and catalysts with vacancy/edge‐hosted N were elaborately synthesized and applied for Fenton‐like reactions. Experimental explorations and DFT calculations suggested that compared with vacancy‐hosted N, the edge‐hosted N exhibited higher catalytic activity for PMS activation and ^1^O_2_ generation. As a result, the edge‐hosted N‐enriched material (N‐Graphene) showed unprecedented PMS activation and pollutant degradation performance, and the remarkable TOF value could even surpass those of single‐atom catalysts. This work provides a deep understanding of the position regulation of active site to greatly improve atom utilization, which can guide the development of efficient Fenton‐like catalysts.

## Conflict of Interest

The authors declare no conflict of interest.

## Author Contributions

H.Z. performed conceptualization, formal analysis, data curation, and wrote the original draft. Z.G. performed investigation and formal analysis. J.Y. performed investigation and formal analysis. Y.H., Q.F., H.Y., and X.X. acquired resources, and performed methodology. Y.T. performed formal analysis and conceptualization. X.C. performed data curation. J.W. performed conceptualization, methodology, supervision, funding acquisition, wrote the original draft, and reviewed and edited the final manuscript. G.O. performed supervision and funding acquisition.

## Supporting information



Supporting Information

## Data Availability

The data that support the findings of this study are available from the corresponding author upon reasonable request.
