# Supplementary material for: Remarkable Active Site Utilization in Edge‐Hosted‐N Doped Carbocatalysts for Fenton‐Like Reaction
Source: Adv Sci (Weinh). 2024 Sep 11;11(41):2404958. doi: 10.1002/advs.202404958 (PMC11538648; doi:10.1002/advs.202404958)
Supplement: Supplementary file 1 — Supporting Information [file ADVS-11-2404958-s001.docx]

**Remarkable active site utilization in edge-hosted-N doped carbocatalysts for Fenton-like reaction**

Huajie Zhong^a^, Zeyu Gong^a^, Jiaxing Yu^b^, Yu Hou^b^, Yuan Tao^b^, Qi Fu^b^, Huangsheng Yang^b^, Xinzhe Xiao^a^, Xingzhong Cao^c^, Junhui Wang^a,^*, Gangfeng Ouyang^a,b,d,e,*^

^a^ School of Chemical Engineering and Technology, Sun Yat-Sen University, Zhuhai 519082 Guangdong, P. R. China

^b^ MOE Key Laboratory of Bioinorganic and Synthetic Chemistry/KLGHEI of Environment and Energy Chemistry, School of Chemistry, Sun Yat-Sen University, Guangzhou, Guangdong, 510275, China

^c^ Institute of High Energy Physics, Chinese Academy of Sciences, Beijing 100049, China

^d^ College of Chemistry & Molecular Engineering, Center of Advanced Analysis and Computational Science, Zhengzhou University, Zhengzhou 450001, China

^e^ Guangdong Provincial Key Laboratory of Emergency Test for Dangerous Chemicals, Guangdong Institute of Analysis (China National Analytical Center Guangzhou), Guangdong Academy of Science ,100 Xianlie Middle Road, Guangzhou 510070, China

* Corresponding author:

Tel: +86 020 84110845/0953; fax: +86 020 84110845/0953.

E-mail address: cesoygf@mail.sysu.edu.cn (Gangfeng Ouyang).

### Text 1. Materials and regents

The following reagents were used as received: G-MWCNT (XF NANO, outer diameter; 10-20 nm, length: 5-30 μm), p-chlorophenol (4-CP), bisphenol A (BPA)，phenol，furfuryl alcohol (FFA), (1,4-Benzoquinone) p-BQ, 2,2’-azino-bis (3-ethylbenzothiazoline-6-sulfonic acid) diammonium salt (ABTS), KI, 2,2,6,6-tetramethyl-4-piperidinol (TEMP, ≥98%), methanol, ethanol, and acetone were purchased from Aladdin. 5,5-dimethyl-1-pyrrolidine N-oxide (DMPO, 98%) was obtained from Dojindo. potassium peroxymonosulfate (PMS, KHSO_5_) was obtained from damas-beta. Phosphate buffer solutions (saline free) were obtained from Yuanye. Nafion solution (5 wt.%) was purchased from Sigma-Aldrich. Ultrapure deionized water (>18 MΩ·cm), produced with a Millipore system, was used for the preparation of all experimental solutions. All chemicals were of reagent grade and were used without further purification or treatment.

### Text 2. Preparation of catalysts

Synthesis of E-CNT

E-CNT was fabricated from a G-MWCNT by plasma treatment (NE-PE02, Shenzhen Naen Tech Co., Ltd, China) for 60 minutes under an atmosphere of argon at 300 W.

Synthesis of V-CNT

V-CNT was fabricated from a G-MWCNT by plasma treatment for 10 minutes under an atmosphere of argon at 300 W.

Synthesis of E-N-CNT

The E-CNT was annealed at 700°C for 3 h in the ammonia flow to obtain E-N-CNT.

Synthesis of V-N-CNT

The V-CNT was annealed at 700°C for 3 h in the ammonia flow to obtain V-N-CNT.

Synthesis of N-CNT-x

N-CNT-x was fabricated from a G-MWCNT by plasma treatment for x minutes under an atmosphere of argon at 300 W, and the defect CNT was annealed at 700°C for 3 h in the ammonia flow to give N-CNT-x.

Synthesis of N-Graphene

The graphene (XF NANO, tablet diameter: 0.5-5 μm, thickness: 0.8 nm) was annealed at 700°C for 3 h in the ammonia flow to obtain N-Graphene.

### Text 3. Characterization

Raman spectra were recorded on a Renishaw InVia spectrometer with a model 100 Ramascope optical fiber instrument. X-ray photoelectron spectroscopic (XPS) analysis was conducted on an AXIS SUPRA+ (Kratos Analytical Co. LTD.) with Al Kα radiation as the exciting source (250 W). The binding energies of the recorded XPS spectra were corrected according to the C 1s line at 284.8 eV. The N-CNTs were inspected using a high-resolution transmission electron microscope (JEM-2010HR, JEOL, Japan). The positron annihilation lifetime spectroscopy measurements (PALS) were performed at room temperature using a conventional fast-slow coincidence system. A positron source was sandwiched between the membranes that had been stacked to a thickness of ~0.8 mm. The γ-rays with energies of 1.27 MeV (emitted from a β-decay of 22Na) and 0.511 MeV (emitted from positron annihilation in a sample) were measured by the start and stop counters, respectively. Each spectrum had ~6 million counts and a time resolution of ~210 ps full width at half-maximum (fwhm). The lifetime spectrum (with total counts of more than 2 million) was analyzed through a finite-term lifetime analysis, which was resolved into four discrete components using the LT9.0 program. The electron paramagnetic resonance spectroscopy measurements (EPR) were obtained on a JESFA-200 (JEOL, Japan) spectrometer. Brunauer-Emmett-Teller (BET) surface areas were investigated by nitrogen adsorption and desorption at liquid nitrogen temperature (77K) using a volumetric adsorption analyzer (BSD-660, BSD, China). Powder X-ray diffraction (PXRD) patterns were collected (0.02°/step, 0.12 seconds/step) on a Bruker D8 Advance diffractometer (Cu Kα) at room temperature. The potential of N-CNTs-PMS complexes was measured on a CHI 660E electrochemical workstation via chronopotentiometry analysis. It was operated in a three-electrode system, Ag/AgCl electrode as the reference electrode, Pt wire as the counter electrode, 50 mM Na_2_SO_4_ solution (100 mL) as electrolyte solution. 1 mL PMS (0.15 M) and 2.5 mL 4-CP (400 ppm) was added into solution to changing the potential of N-CNTs when the potential of N-CNTs was stable. The potential of N-CNTs-PMS complexes represents the stable equilibrium potential of N-CNTs after adding PMS.

### Text 4. Activity evaluation

The experiments were carried out in 50 ml glass reactors at room temperature. The pH value of the entire reaction system was not further adjusted by any buffers. The reaction solution was prepared by suspending CNTs (2 mg) in 20 mL of 4-CP solution (C_0_=0.08 mM). After reaching the adsorption equilibrium, the reaction was initiated by adding 0.2 mL prepared solution of PMS (150 mM). Then 0.5 mL samples were withdrawn using a 1 mL syringe at certain intervals during the 20 minutes reaction, and the solid was removed by filtration (0.22 μm PTEF filter). The concentration of 4-CP was analyzed using a HPLC (Shimadzu LC-20AD). Separation was performed on a Poroshell 120 EC-C18 column (4.6×100 mm, 2.7 μm, Agilent Technology, USA) using a mobile phase consisting of a binary mixture of water and methanol at a flow rate of 0.16 mL/min and 0.48 mL/min individually.

The concentration of PMS was measured by the ABTS colorimetric method. The ABTS colorimetric method is the concentration of PMS was determined based on a previously proposed 2,2’-azino-bis (3-ethylbenzothiazoline-6-sulfonic acid) diammonium salt (ABTS) colorimetric method. The detailed experimental procedures are as follows. Briefly, 0.5 mL filtered sample was added into a 10 mL colorimetric tube, followed by successive addition of 1 mL pH=4 acetic buffer solution, 20 μL KI solution (1.5 mM), and 1 mL ABTS solution (2 mM), and then was diluted with ultrapure water. The spectrophotometry was performed at a detection wavelength of 415nm.

### Text 5. Density functional theory (DFT) calculations

All the calculations are performed in the framework of the density functional theory with the projector augmented plane-wave method, as implemented in the Vienna ab initio simulation package ^[1]^. The generalized gradient approximation proposed by Perdew, Burke, and Ernzerhof is selected for the exchange-correlation potential ^[2]^. Weak van der Waals interaction is considered by the DFT-D3 functional ^[3]^. The cutoff energy for the plane wave is set to 400 eV. The energy criterion is set to 10^−5^ eV in the iterative solution of the Kohn-Sham equation. The Brillouin zone integration is performed at the Gamma point. All the structures are relaxed until the residual forces on the atoms have declined to less than 0.05 eV/Å. In this work, single wall carbon nanotubes are taken as an example to consider the catalytic effect for persulfate adsorption and activation. The adsorption energy is studied by DFT calculations, defined as:

E_ads_= E_total_ - E_substrate_ - E_molecule_

Where E_total_, E_substrate_, and E_molecule_ denote the total energy of substrate with adsorbate, substrate, and free molecule, respectively.

In all experiments performed, no unexpected or unusually high safety hazards were encountered.


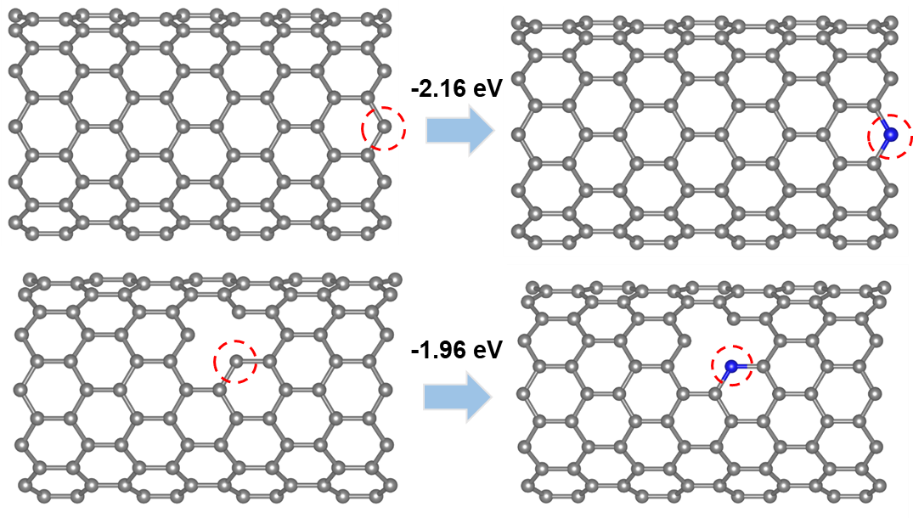


**Figure S1.** Two reaction pathways that form specific N doping carbon catalyst with vacancy and edge defects.

**Figure S2.** XRD patterns of CNT, E-N-CNT and V-N-CNT.

**Figure S3.** The C K-edge XANES spectra.

**
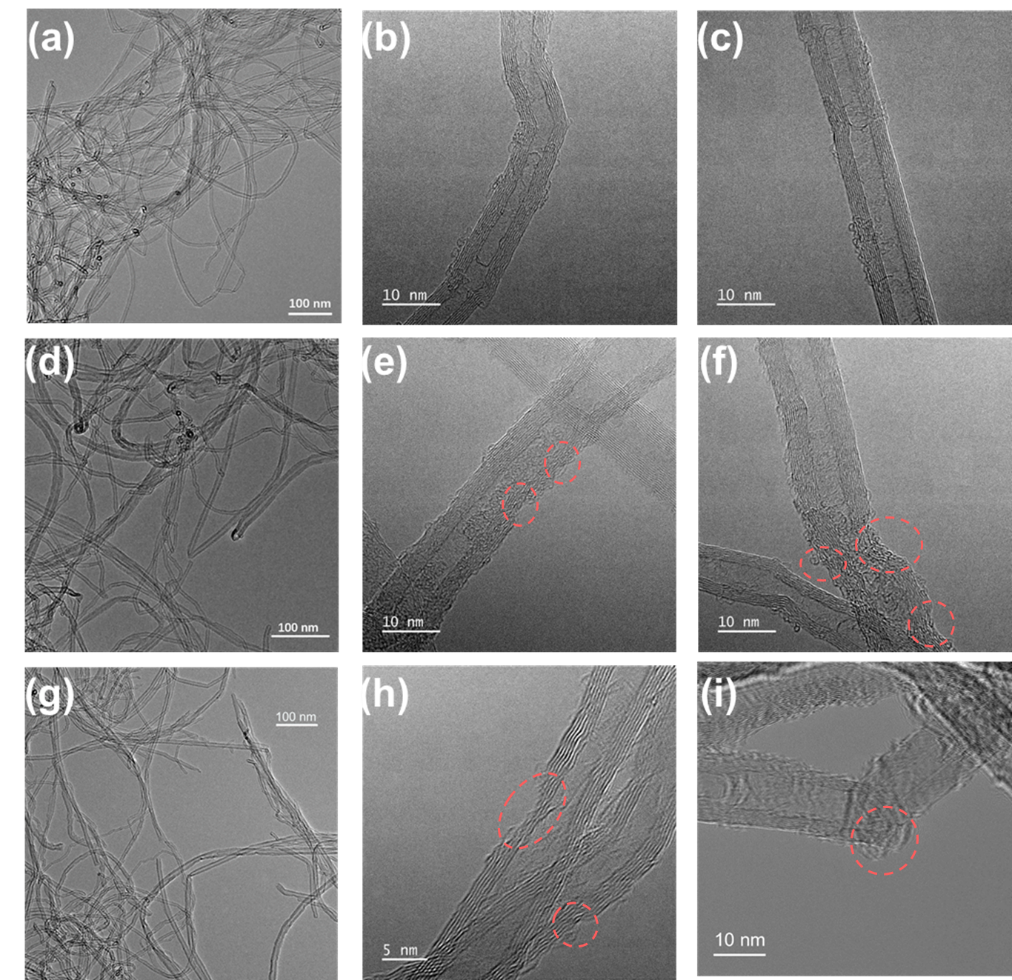
**

**Figure S4.** HRTEM images for CNT (a, b, c), V-N-CNT (d, e, f) and E-N-CNT (g, h, i). Red circle regions in images represent the defect sites etched by plasma.

**Figure S5.** PALS spectra of E-N-CNT, V-N-CNT and CNT.

**Figure S6.** Raman spectra of E-N-CNT and E-CNT.

**Figure S7.** 4-CP degradation on E-N-CNT and E-CNT-700.

**Figure S8.** The pseudo first-order kinetic model fitting of E-N-CNT, V-N-CNT, E-CNT, V-CNT and PMS.

**Figure S9.** N_2_ adsorption-desorption isotherms of CNT, E-N-CNT and V-N-CNT.

**Figure S10.** 4-CP adsorption on E-N-CNT and V-N-CNT. Reaction conditions: catalyst dosage = 0.1 g L^-1^, [4-CP] = 10 ppm.

**Figure S11.** The Raman spectra of different N-CNT catalysts.

**
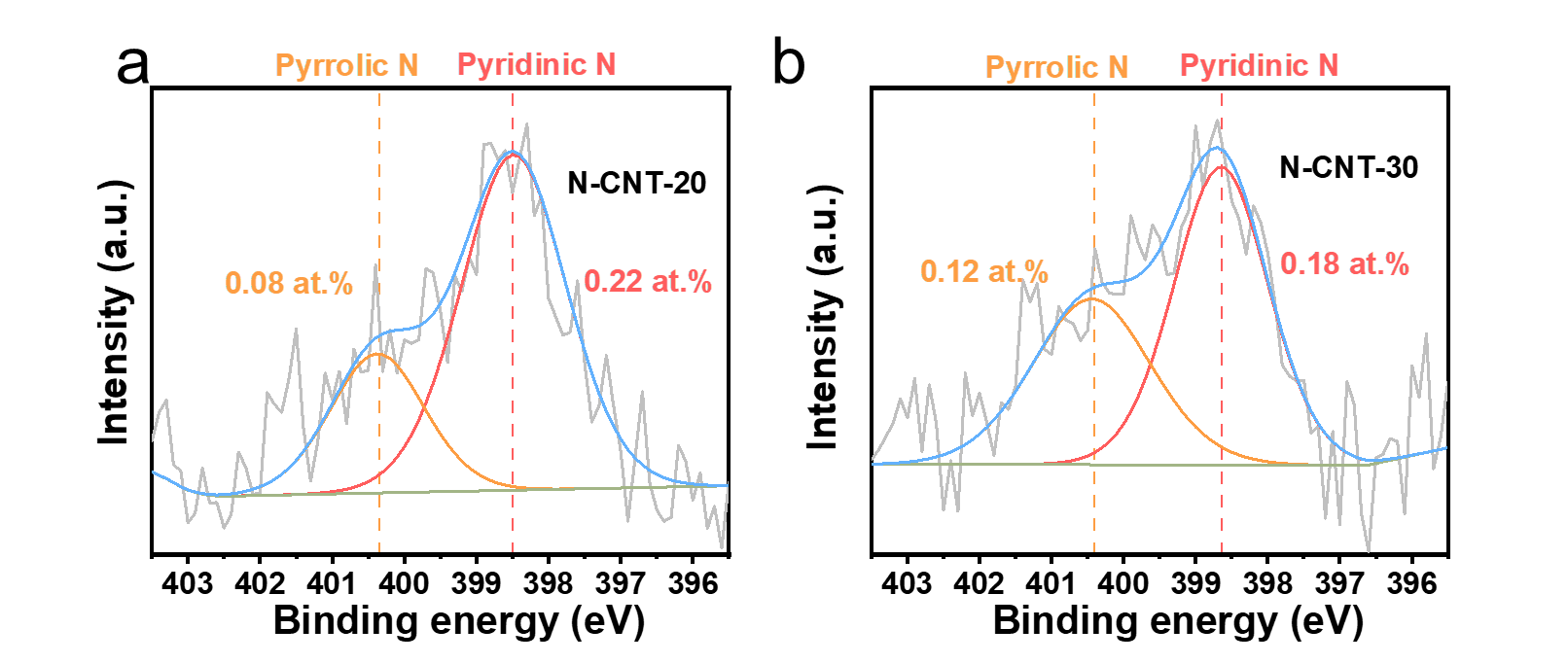
**

**Figure S12.** N 1s XPS spectra of (a) N-CNT-20 and (b) N-CNT-30.

**Figure S13.** 4-CP degradation on different N-CNT catalysts. Reaction conditions: catalyst dosage = 0.1 g L^-1^, [PMS] = 1.5 mM, [4-CP] = 10 ppm.

**Figure S14.** The pseudo first-order kinetic model fitting of N-CNTs.

**Figure S15.** N 1s XPS spectra of N-Graphene.

**
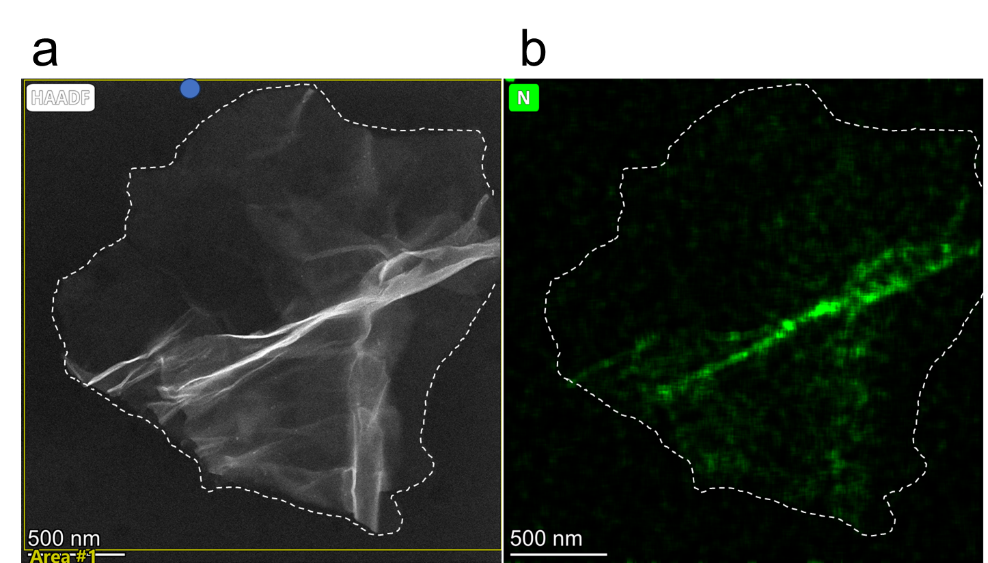
**

**Figure S16.** (a) HAADF-HRTEM image and (b) the corresponding EDS mapping of N-Graphene.

**Figure S17.** 4-CP degradation on N-Graphene and Graphene in the presence of PMS. Reaction conditions: catalyst dosage = 0.1 g L^-1^, [PMS] = 1.5 mM, [4-CP] = 10 ppm.

**Figure S18.** The kinetic analysis of 4-CP degradation on N-Graphene and Graphene in the presence PMS according to pseudo-first-order model.

**Figure S19.** The kinetic analysis of 4-CP degradation by the E-N-CNT/PMS system in the presence of diverse scavengers according to pseudo-first-order model.

**Figure S20.** EPR spectra obtained by spin trapping with DMPO (water as the solvent).

**Figure S21.** EPR spectra obtained by spin trapping with TEMP in the presence of 4-CP in the E-N-CNT/PMS system.

**Figure S22.** EPR spectra obtained by spin trapping with DMPO. (methanol as the solvent).

**Figure S23.** EPR spectra obtained by spin trapping with TEMP.

**Figure S24.** Effect of inorganic anions on 4-CP degradation. Reaction conditions: catalyst dosage = 0.1 g L^-1^, [PMS] = 1.5 mM, [4-CP] = 10 ppm, [Cl^-^] = [SO_4_^2-^] = [HCO_3_^-^] = [NO_3_^-^] =10 mM.

**Figure S25.** 4-CP removal efficiency of E-N-CNT, used E-N-CNT and regenerated E-N-CNT. Reaction conditions: catalyst dosage = 0.1 g L^-1^, [PMS] = 1.5 mM, [4-CP] = 10 ppm, reaction time = 20 min.

**
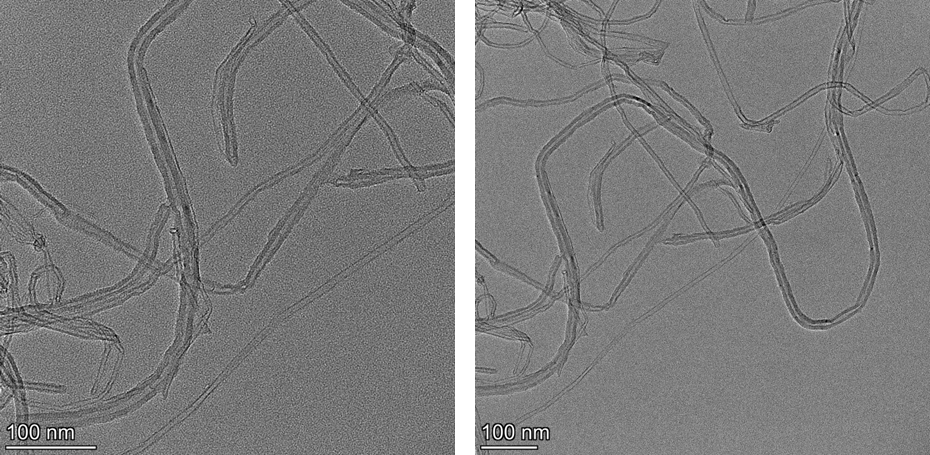
**

**Figure S26.** TEM images of used E-N-CNT.

**Figure S27.** Raman spectra of E-N-CNT and used E-N-CNT.

**
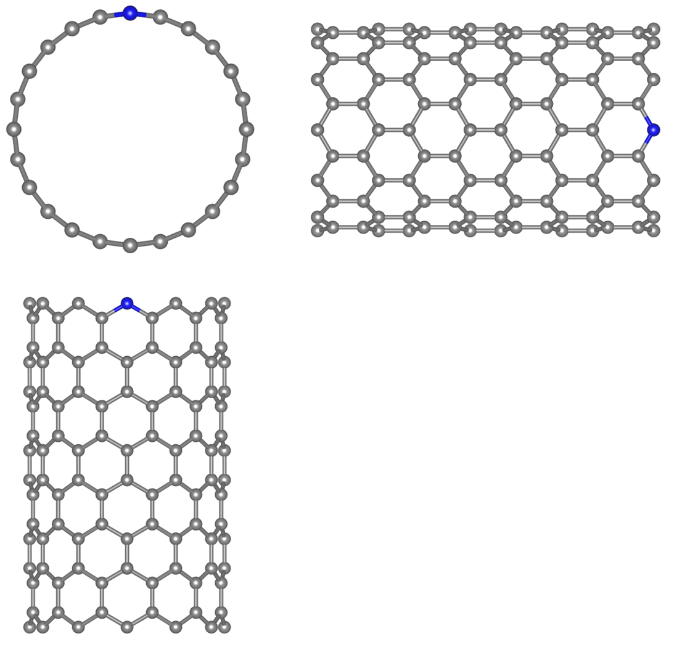
**

**Figure S28.** The structure of E-N-CNT from different views.


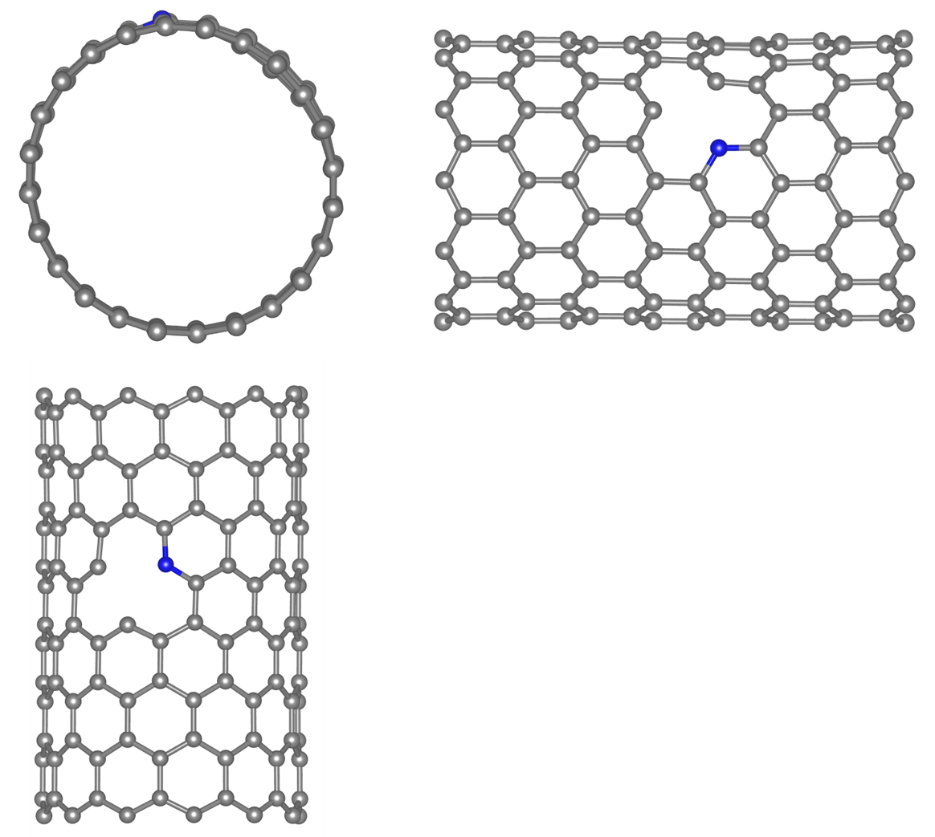


**Figure S29.** The structure of V-N-CNT from different views. One C atom was removed according to the PALS analysis.

**
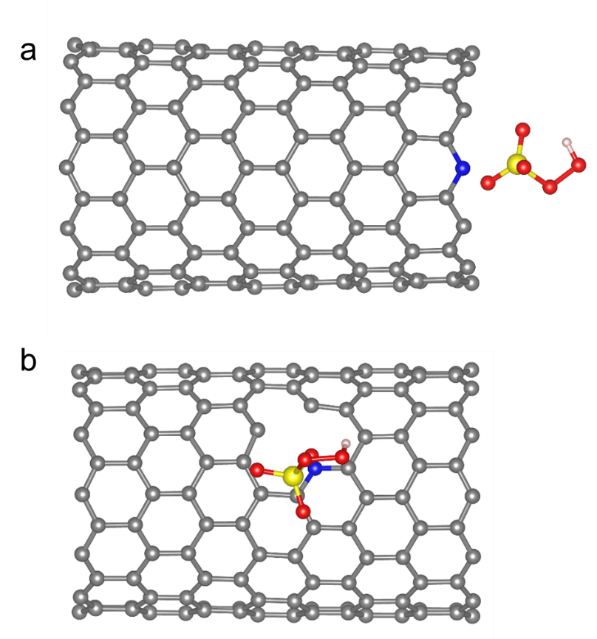
**

**Figure S30.** Adsorption models of PMS on the N sites of E-N-CNT (a) and V-N-CNT (b).

**Figure S31.** Adsorption energies of PMS on different sites of E-N-CNT and V-N-CNT.

**Figure S32.** In-situ Raman spectra of E-N-CNT/PMS over time.

**
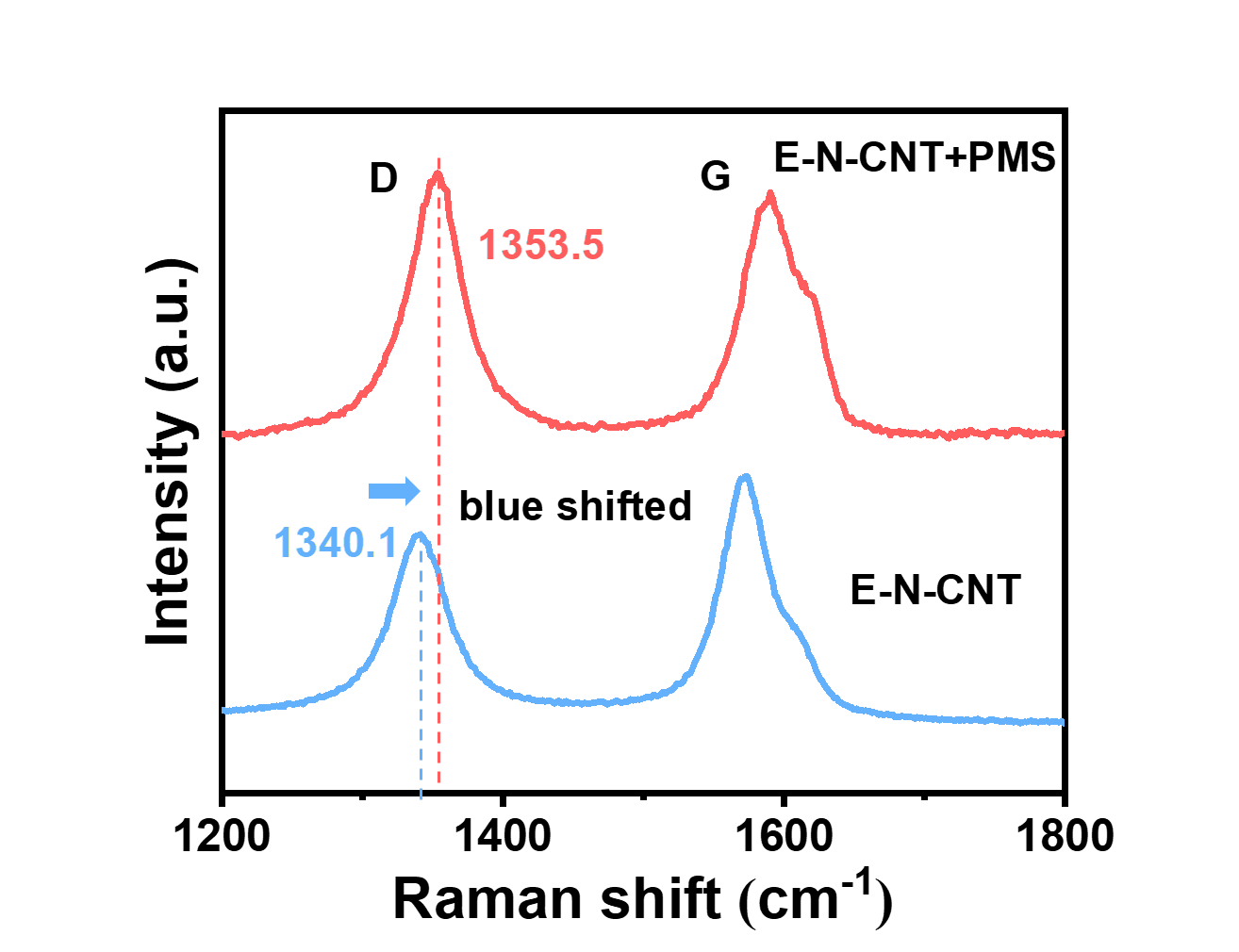
**

**Figure S33.** In-situ Raman spectra of E-N-CNT and E-N-CNT/PMS.

| Sample | Lifetime 1 (ns) | Intensity 1 | Lifetime 2 (ns) | Intensity 2 | Lifetime 3 (ns) | Intensity 3 |
| --- | --- | --- | --- | --- | --- | --- |
| CNT | 0.1805 | 77.08 | 0.4199 | 21.6 | 3.2 | 1.327 |
| V-N-CNT | 0.1791 | 79.19 | 0.4320 | 19.56 | 3.022 | 1.257 |
| E-N-CNT | 0.1635 | 77.9 | 0.3542 | 20.8 | 2.248 | 1.29 |

**Table S1**. The lifetime of positron on CNT, V-N-CNT and E-N-CNT.

**Table S2**. Comparison of TOF values of organic contaminants degradation in typical reported Fenton-like processes based on N-doped metal-free carbon catalysts.

| No. | Catalysts  (g L^-1^) | Pollutant  (mM) | PMS  (mM) | Removal Efficiency | *k*  (min^-1^) | *k*-value  (L min^-1^ g^-1^) | N Loading  (at. %) | TOF  (min^-1^) | Ref. |
| --- | --- | --- | --- | --- | --- | --- | --- | --- | --- |
| 1 | PCN-0.5 (0.05) | 4-CP (0.160) | 1.00 | 100%  (2 min) | 1.450 | 4.6400 | 18.50 | 0.1211 | ^[6]^ |
| 2 | NBC700 (0.5) | 4-CP (0.8) | 3.25 | 61.4% (180 min) | 0.023 | 0.0111 | 3.51 | 0.0022 | ^[12]^ |
| 3 | NoCNT-700 (0.1) | Phenol (0.210) | 6.50 | 100%  (20 min) | 0.247 | 0.0797 | 0.80 | 0.1838 | ^[20]^ |
| 4 | NPCZIF-8 (0.2) | Phenol (0.210) | 1.60 | 100%  (60 min) | 0.079 | 0.0518 | 5.50 | 0.0045 | ^[21]^ |
| 5 | P-NCC-0.8 (0.05) | Phenol (0.210) | 2.26 | 100%  (10 min) | 1.510 | 0.9653 | 4.14 | 0.1420 | ^[22]^ |
| 6 | C_N_-1000-0.1 (0.1) | Phenol (0.210) | 1.00 | 100%  (5 min) | 0.696 | 1.4616 | 2.16 | 0.2722 | ^[23]^ |
| 7 | CPANI-9 (0.025) | Phenol (0.010) | 0.50 | 100%  (10 min) | 0.347 | 0.2776 | 6.16 | 0.0091 | ^[24]^ |
| 8 | N-C-6  (0.4) | Phenol (0.106) | 0.20 | 98%  (30 min) | 0.110 | 0.1458 | 28.27 | 0.0004 | ^[25]^ |
| 9 | N-CNT-60  (0.2) | Phenol (0.210) | 1.00 | 100%  (30 min) | 0.127 | 0.1332 | 1.60 | 0.0306 | ^[26]^ |
| 10 | NCN-900 (0.1) | BPA (0.100) | 2.00 | 100%  (2 min) | 3.100 | 1.5500 | 14.50 | 0.0483 | ^[27]^ |
| 11 | NC1.0 (0.2) | BPA (0.100) | 2.00 | 100%  (15 min) | 0.260 | 0.0650 | 33.75 | 0.0014 | ^[28]^ |
| 12 | NOPC (0.2) | BPA (0.044) | 0.15 | 100%  (20 min) | 0.300 | 0.4368 | 40.00 | 0.0004 | ^[29]^ |
| 13 | NOPC-700 (0.125) | BPA  (0.088) | 0.65 | 63.5%  (15 min) | 0.177 | 0.1908 | 7.15 | 0.0058 | ^[30]^ |
| 14 | NC900 (0.04) | BPA  (0.088) | 2.63 | 81%  (20 min) | 0.075 | 0.0625 | 20.68 | 0.0060 | ^[31]^ |
| 15 | NCs  (0.15) | BPA (0.044) | 0.40 | 100%  (60 min) | 0.127 | 0.0927 | 17.93 | 0.0004 | ^[32]^ |
| 16 | N-CM900 (0.1) | 2,4-DCP (1.227) | 1.97 | 100%  (30 min) | 0.150 | 0.9343 | 17.91 | 0.0320 | ^[33]^ |
| 17 | NPC-700 (0.1) | MB (0.156) | 2.00 | 99.15%  (30 min) | 0.283 | 0.2206 | 8.25 | 0.0087 | ^[34]^ |
| 18 | N-C-8  (0.2) | MB (0.063) | 0.99 | 99%  (60 min) | 0.083 | 0.0263 | 5.50 | 0.0013 | ^[35]^ |
| 19 | N-MPGC-2 (1.0) | RhB (0.042) | 1.00 | 100%  (15 min) | 0.293 | 0.0123 | 5.00 | 0.0008 | ^[36]^ |
| 20 | E-N-CNT (0.1) | 4-CP  (0.080) | 1.5 | 100%  (7 min) | 0.558 | 0.2973 | 0.29 | 0.5517 | This |
| 21 | V-N-CNT (0.1) | 4-CP  (0.080) | 1.5 | 70.7%  (20 min) | 0.060 | 0.0317 | 0.28 | 0.1414 |  |
| 22 | N-Graphene (0.1) | 4-CP  (0.080) | 1.5 | 100%  (10 s) | 20.116 | 10.7285 | 1.76 | 3.8182 | work |

The turnover frequency (TOF, min^−1^) of N site, which represents the catalyst’s intrinsic activity and the utilization of nitrogen atom can be calculated by eq. S1^[37]^.

TOF = $\frac{\text{∆}\text{n(pollutants)×}\text{M}_{\text{nitrogen}}}{\text{m}_{\text{0}}\text{×}\text{ω}_{\text{nitrogen}}\text{×t}}$ (S1)

Where $\text{∆}\text{n(pollutants)}$ is the moles of pollutants converted, $\text{M}_{\text{nitrogen}}$ is the atomic weight of nitrogen, $\text{m}_{\text{0}}$ is the mass dosing of catalysts, $\text{ω}_{\text{nitrogen}}$ is the atomic concentration of nitrogen in catalysts, $\text{t}$ is the reaction time.

**Table S3.** Comparison of the *k*-value of 4-CP degradation in typical reported Fenton-like processes based on PMS.

| **No.** | **Catalysts** | **Dosage**  **(g L^-1^)** | **4-CP**  **(mM)** | **PMS**  **(mM)** | **Removal**  **Efficiency** | ***k***  **(min^-1^)** | ***k*-value**  **(L min^-1^ g^-1^)** | **Ref.** |
| --- | --- | --- | --- | --- | --- | --- | --- | --- |
| 1 | CNF_3_ | 0.10 | 0.10 | 1.00 | 100% (20 min) | 0.254 | 0.254 | ^[4]^ |
| 2 | Fe_3.75_-CN/G_5.0_ | 0.10 | 0.10 | 1.00 | 100% (10 min) | 0.495 | 0.495 | ^[5]^ |
| 3 | PCN-0.5 | 0.05 | 0.16 | 1.00 | 100% (2 min) | 1.45 | 4.640 | ^[6]^ |
| 4 | o-CNTs-1000 | 0.10 | 0.16 | 0.50 | 100% (60 min) | 0.0955 | 0.306 | ^[7]^ |
| 5 | CoO-N-C | 0.30 | 0.40 | 1.50 | 100% (40 min) | 0.17 | 0.151 | ^[8]^ |
| 6 | CuCo@ZnO | 0.20 | 0.32 | 2.00 | 96% (40 min) | 0.036 | 0.029 | ^[9]^ |
| 7 | CFNC-30 | 0.09 | 0.40 | 7.23 | 100% (20 min) | 0.241 | 0.150 | ^[10]^ |
| 8 | MS-VO-Co_3_O_4_ | 0.05 | 0.40 | 1.32 | 98.4% (20 min) | 0.186 | 1.132 | ^[11]^ |
| 9 | NBC800 | 0.50 | 0.80 | 3.25 | 90% (180 min) | 0.0375 | 0.018 | ^[12]^ |
| 10 | CoO-A | 0.03 | 0.08 | 0.25 | 99.2% (10 min) | 0.428 | 4.565 | ^[13]^ |
| 11 | FCCN_V_ | 0.15 | 0.40 | 4.93 | 100% (30 min) | 0.227 | 0.123 | ^[14]^ |
| 12 | Fe/Fe_3_O_4_@CPPy-4 | 0.08 | 0.016 | 0.25 | 98.64% (10 min) | 0.392 | 0.314 | ^[15]^ |
| 13 | CuO | 0.20 | 0.08 | 0.20 | 100% (3 min) | 2.2 | 4.400 | ^[16]^ |
| 14 | Fe-MOF@Mn_2_O_3_ | 0. 20 | 0.08 | 2.00 | 91.4% (60 min) | 0.086 | 0.017 | ^[17]^ |
| 15 | Fe_3_C/Mo_2_C@CPANI-0.1 | 0.03 | 0.032 | 0.40 | 98.27% (10 min) | 0.79 | 2.107 | ^[18]^ |
| 16 | Co-CuO_300_ | 0.15 | 0.20 | 8.28 | 100% (10 min) | 0.7607 | 0.122 | ^[19]^ |
| 17 | E-N-CNT | 0.10 | 0.08 | 1.50 | 100% (7 min) | 0.5575 | 0.297 | This |
| 18 | N-Graphene | 0.10 | 0.08 | 1.50 | 100% (10 s) | 20.116 | 10.728 | work |

The unified kinetic rate (*k*-value) was calculated through dividing *k* by the catalyst dosage (ρ_cat_, g L^−1^) and peroxide concentration (c_0_, mol L^−1^), followed by multiplying initial organic contaminant concentration (c_1_, mol L^−1^) (Eq. S2). ^[38]^

*k*-value = *k* × c_1_/ (c_0_ × ρ_cat_). (S2)

**Table S4.** N contents of different N-CNTs catalysts by XPS.

| **Samples** | **Total N (at%)** | **Pyridinic N**  **(at%)** | **Pyrrolic N**  **(at%)** | **Graphitic N**  **(at%)** | **Oxidized N**  **(at%)** |
| --- | --- | --- | --- | --- | --- |
| V-N-CNT | 0.28 | 0.18 | 0.10 | 0.00 | 0.00 |
| N-CNT-20 | 0.30 | 0.22 | 0.08 | 0.00 | 0.00 |
| N-CNT-30 | 0.30 | 0.18 | 0.12 | 0.00 | 0.00 |
| E-N-CNT | 0.29 | 0.16 | 0.13 | 0.00 | 0.00 |
| Graphene | 0.19 | 0.00 | 0.08 | 0.11 | 0.00 |
| N-Graphene | 1.76 | 0.79 | 0.47 | 0.25 | 0.25 |
| Used E-N-CNT | 0.31 | 0.19 | 0.12 | 0.00 | 0.00 |

**Table S5.** Elemental content of C, N, O and Cl from XPS survey spectra.

| **Samples** | **C**  **(at%)** | **N**  **(at%)** | **O**  **(at%)** | **Cl**  **(at%)** |
| --- | --- | --- | --- | --- |
| V-N-CNT | 98.82 | 0.28 | 0.90 | / |
| N-CNT-20 | 97.61 | 0.30 | 2.09 | / |
| N-CNT-30 | 98.59 | 0.29 | 1.11 | / |
| E-N-CNT | 98.41 | 0.29 | 1.29 | / |
| E-CNT | 98.59 | / | 1.41 | / |
| E-N-CNT treated by PMS | 98.61 | 0.20 | 1.19 | / |
| used E-N-CNT | 96.49 | 0.31 | 2.54 | 0.66 |
| Graphene | 91.26 | 0.19 | 8.55 | / |
| N-Graphene | 92.49 | 1.76 | 5.75 | / |

[1] G. Kresse, J. Furthmüller, *Phys. Rev. B* **1996**, *54*, 11169-11186.

[2] J. P. Perdew, K. Burke, M. Ernzerhof, *Phys. Rev. Lett.* **1996**, *77*, 3865-3868.

[3] S. Grimme, J. Antony, S. Ehrlich, H. Krieg, *J. Chem. Phys.* **2010**, *132*, 154104.

[4] H. Li, C. Shan, B. Pan, *Environ. Sci. & Technol.* **2018**, *52*, 2197-2205.

[5] H. Li, C. Shan, B. Pan, *Sci. Total. Environ.* **2019**, *675*, 62-72.

[6] J. Miao, W. Geng, P. J. J. Alvarez, M. Long, *Environ. Sci. Technol.* **2020**, *54*, 8473-8481.

[7] P. Shao, S. Yu, X. Duan, L. Yang, H. Shi, L. Ding, J. Tian, L. Yang, X. Luo, S. Wang, *Environ. Sci. Technol.* **2020**, *54*, 8464-8472.

[8] M. Xie, J. Tang, G. Fang, M. Zhang, L. Kong, F. Zhu, L. Ma, D. Zhou, J. Zhan, *J. Hazard. Mater.* **2020**, *384*, 121345.

[9] X. Zhou, C. Luo, M. Luo, Q. Wang, J. Wang, Z. Liao, Z. Chen, Z. Chen, ***Chem. Eng. J.*** **2020**, *381*, 122587.

[10] Y. Zhou, Y. Zhang, X. Hu, ***J. Colloid Interface Sci****.* **2020**, *575*, 206-219.

[11] P. Li, Y. Lin, S. Zhao, Y. Fu, W. Li, R. Chen, S. Tian, ***Appl. Catal. B*** **2021**, *298*, 120596.

[12] Z. Wan, Z. Xu, Y. Sun, M. He, D. Hou, X. Cao, D. C. W. Tsang, *Environ. Sci. Technol.* **2021**, *55*, 7004-7014.

[13] Q. Wang, Z. Xu, S. Wang, Z. Wang, J. Jia, H. Li, Y. Cao, Y. Chen, Y. Qin, F. Cui, Sep. Purif. Technol. **2021**, *263*.

[14] Y. Zhou, X. Hu, Y. Zhang, X. Chen, H. Zhao, Q. Fu, F. Xu, Y. Gao, *Appl. Surf. Sci.* **2021**, *566*, 150657.

[15] Q. Wang, Y. Jiang, S. Yang, J. Lin, J. Lu, W. Song, S. Zhu, Z. Wang, ***Chem. Eng. J.*** **2022**, *445*, 136806.

[16] Y. Wei, J. Miao, J. Ge, J. Lang, C. Yu, L. Zhang, P. J. J. Alvarez, M. Long, *Environ. Sci. Technol.* **2022**, *56*, 8984-8992.

[17] Y. Xue, L. Liu, T. Zhang, Z. Zhu, M. Xu, Q. Zhang, J. Hong, *J. Environ. Chem. Eng.* **2022**, *10*, 108522.

[18] Q. Wang, Y. Jiang, X. Lin, H. Li, Z. Wang, W. Wang, Sep. Purif. Technol. **2023**, *322*, 124359.

[19] L. Zhou, P. Li, X. Yang, J. Wu, B. Hou, B. Xu, X. Liu, K. Zhang, W. Jiang, Sep. Purif. Technol. **2023**, *325*, 124671.

[20] X. Duan, H. Sun, Y. Wang, J. Kang, S. Wang, *ACS Catal.* **2015**, *5*, 553-559.

[21] G. Wang, S. Chen, X. Quan, H. Yu, Y. Zhang, *Carbon* **2017**, *115*, 730-739.

[22] J. Wang, Z. Yang, Y. Li, X. Fan, F. Zhang, G. Zhang, W. Peng, S. Wang, *Carbon* **2020**, *163*, 43-55.

[23] Y. Zhang, H. Pan, M. Murugananthan, P. Sun, D. D. Dionysiou, K. Zhang, A. Khan, Y. Zhang, *Carbon* **2020**, *156*, 399-409.

[24] S. Liu, Z. Zhang, F. Huang, Y. Liu, L. Feng, J. Jiang, L. Zhang, F. Qi, C. Liu, ***Appl. Catal. B*** **2021**, *286*, 119921.

[25] Y. Wang, Z. Zhang, Z. Yin, Z. Liu, Y. Liu, Z. Yang, W. Yang, ***Appl. Catal. B*** **2022**, *319*.

[26] S. Liu, S. Yin, Z. Zhang, L. Feng, Y. Liu, L. Zhang, *J. Hazard. Mater.* **2023**, *441*, 129905.

[27] Y. Gao, Z. Chen, Y. Zhu, T. Li, C. Hu, *Environ. Sci. Technol.* **2020**, *54*, 1232-1241.

[28] Y. Gao, T. Li, Y. Zhu, Z. Chen, J. Liang, Q. Zeng, L. Lyu, C. Hu, *J. Hazard. Mater.* **2020**, *393*, 121280.

[29] Y.-L. He, C.-S. He, L.-D. Lai, P. Zhou, H. Zhang, L.-L. Li, Z.-K. Xiong, Y. Mu, Z.-C. Pan, G. Yao, B. Lai, ***Appl. Catal. B*** **2022**, *314*, 121390.

[30] B. Lu, F. Wu, Z. Yin, C. He, J. Qian, Y. Mao, X. You, G. Lin, X. Yang, B. Huang, *Appl. Surf. Sci.* **2023**, *640*, 158308.

[31] S. Wang, Y. Xia, L. Tan, S. Wu, Y. Yu, X. Yu, Z. Guan, H. Chen, F. Jiang, Sep. Purif. Technol. **2023**, *317*, 123873.

[32] G. Qu, P. Jia, S. Tang, M. N. Pervez, Y. Pang, B. Li, C. Cao, Y. Zhao, *J. Hazard. Mater.* **2024**, *461*, 132626.

[33] J. Xu, P. Zhou, P. Shi, Y. Min, Q. Xu, *J. Environ. Chem. Eng.* **2021**, *9*, 106545.

[34] Y. Hu, D. Chen, S. Wang, R. Zhang, Y. Wang, M. Liu, Sep. Purif. Technol. **2022**, *280*, 119791.

[35] H. Wang, H. Wang, Q. Yan, *Sci. Total. Environ.* **2022**, *816*, 151611.

[36] K. Zhu, C. Liu, W. Xia, Y. Wang, H. He, L. Lei, Y. Ai, W. Chen, X. Liu, *J. Colloid Interface Sci.* **2022**, *625*, 890-902.

[37] P. Yang, Z. Cao, Y. Long, D. Liu, W. Huang, S. Zhan, M. Li, *ACS Catal.* **2023**, *13*, 12414-12424.

[38] X. Yu, H. Liu, Y. Huang, C. Li, L. Kuang, J. Zhong, S. Zhu, Y. Gou, Y. Wang, Y. Zhang, G. Shan, Z. Lv, S. Zhang, L. Zhu, *Proc. Natl. Acad. Sci USA* **2023**, 120, e2221228120.
